# A Review of Developmental Scales in Pediatric Practice: Recent Guidelines

**DOI:** 10.7759/cureus.62941

**Published:** 2024-06-23

**Authors:** Anuja Handargule, Revat J Meshram, Amar Taksande, Aashita Malik, Sri Sita Naga Sai Priya K, Kushal Desai

**Affiliations:** 1 Pediatrics, Datta Meghe Institute of Higher Education and Research Centre, Wardha, IND

**Keywords:** merrill-palmer-revised scales, autism spectrum disorder, baroda developmental screening test, developmental scales, trivandrum developmental screening chart

## Abstract

Imitation, fine motor abilities, eye-to-hand coordination, perception, gross motor abilities, mental abilities, and verbal cognitive abilities are assessed on the developmental scale. The behavioral scale also assesses social interaction, emotional expression, activity, curiosity, sensory reactivity, and language. The current developmental scales in pediatrics are discussed in this paper. These scales have evolved. International scales for Indian children are difficult to administer due to cultural differences in self-care and gender roles. If parental awareness and demand are raised, postnatal growth interventions for psychosocial development will benefit infants in developing nations. Routine screening involves identifying an appropriate opportunity, acquisition, tool selection, administration, interpreting data, scoring, counseling, and training.

## Introduction and background

Development is an ongoing and natural process that typically takes place mostly during childhood and continues for several years after childhood. During childhood, individuals acquire skills in several interconnected areas of development. It is carefully shaped by a complex interplay of genetic, biological, environmental, and psychosocial variables [1]. Parents often express worries to pediatricians about their child's conduct or growth [2,3]. Some of these abnormalities are temporary and additionally easily rectified, while a few significant numbers may potentially indicate the presence of neuro-developmental diseases. The American Academy of Pediatrics (AAP) has recommended implementing systematic screening along with surveillance to enhance the detection of juvenile developmental and behavioral abnormalities. To improve scrutiny, the AAP recommends that pediatricians inquire, "Do you have any apprehensions regarding the development of your child?" that include behavior and acquiring knowledge or skills [4].

Although the theoretical underpinnings of developmental scales are up for debate, developmental milestones often serve as their basis. At specified phases of development, children exhibit specific developmental milestones. Differences in frequency or style can indicate immaturity or neurological problems, but this is not always the case. As an illustration, the typical age at which children start to stand upright is approximately 12 months. It is important to note that some infants may start to stand as early as 10 months, while others might not start standing until 16 months. This variation in timing does not necessarily imply any developmental issues. These disparities could be seen as a potential risk indicator to monitor the child's growth, or they may be rectified naturally as the youngster grows. Notable milestones for each specific age group and area of development are identified and transformed into individual items. These items constitute the scales [5].

The developmental scale assesses eye-hand coordination, imitation ability, perception, and cognitive functioning, as well as gross motor skills and verbal cognitive abilities. Furthermore, the behavioral scale evaluates other domains such as social interaction and emotional expression, engagement in activities and curiosity towards objects, sensory reactivity, and linguistic abilities [6].

## Review

Merrill-Palmer revised scales of development

The latest iteration of the first Merrill-Palmer scales introduced in 1931, referred to as the Merrill-Palmer-R, continues to employ interactive and hands-on activities that effectively captivate even the youngest participants. Novel, play-thing-oriented tasks evaluate visual-motor skills, cognitive abilities, and problem-solving aptitude by employing safe, vibrant materials. Stoelting has thoroughly modernized, edited, and enlarged the renowned Merrill-Palmer scales of development, following the Leiter international performance scale revised (Leiter-R) tradition [7].

The Merrill-Palmer revised scales of development (M-P-R) is a standardized assessment tool used to determine the intellectual capacity of preschool-aged children. In their study evaluating the M-P-R's accuracy in measuring mental skills in preschool-aged children with autism spectrum disorder, Dempsey et al. discovered strong contemporaneous validity. The M-P-R's receptive language domain and the preschool language scale fourth edition auditory comprehension subscale were found to have a notable positive correlation [8]. This tool is particularly helpful in evaluating the developmental activities of children between the ages of one month and six and a half, as well as infants who were born prematurely. With unique scores allocated to each of the following: cognitive, language/communication, motor (fine and gross), social-emotional development, and self-help/adaptive, the assessment provides a thorough review of several IDEA domains [7].

Bayley scales of infant and toddler development

Early infants with developmental issues can be identified with the thorough, standardized Bayley scales of infant and toddler development (BSID) assessment tool. The development of young children is assessed using a norm-referenced assessment, which yields BSID scores. Nancy Bayley initially released the BSID in 1969. The most recent BSID in use is BSID IV, which was introduced in 2019. Compared to BSD III, BSID IV exhibits superior clinical sensitivity and accuracy, as well as greater effectiveness. The ages of 16 days to 42 months are assessed using the BSID III and BSID IV. It typically takes 30 to 70 minutes to finish the exam [9].

The BSID devises appropriate interventions for children with delays in developmental milestones. The BSID assists in the early detection of cognitive impairment. Re-evaluations can be used to track and measure progress over time to personalize the administration and cater to the developmental and educational requirements of a child. It evaluates specific areas of development, such as cognitive impairment, and serves as a research tool for researchers. Pediatric experts have a crucial role in identifying developmental disabilities at an early stage. The BSID aids in the timely notice of developmental delay and facilitates the prompt implementation of early developmental intervention [10].

Brazelton neonatal behavior assessment scale

Brazelton et al. released the neonatal behavior assessment scale (NBAS) in 1973 as a resource to aid parents, healthcare professionals, and researchers in comprehending the behavioral communication of newborns from birth to two months old. The measure consists of 28 behavioral and 18 reflex items. These items do not provide a numerical score but rather explain several developmental aspects and how the baby combines these aspects in response to environmental stimuli. Brazelton posits that babies encounter a hierarchical structure consisting of four fundamental developmental activities, all of which are assessed by his scale [11].

The NBAS assists parents and other caregivers in learning about their child's development as well as their neurobehavior. Furthermore, parents and other caregivers may decide what their infant requires and the best ways to interact with them. When a preterm neonate is hospitalized and the mother-child bond becomes difficult, the NBAS may be able to enhance caregiver-infant interactions [12].

However, a systematic analysis employing the Cochrane approach was carried out by Barlow et al. [13] to assess the effect of the NBAS and newborn behavioral observations (NBO) systems on improving the caregiver-infant bond and related outcomes in both caregivers and newborns. The study's secondary goals were to evaluate the NBAS and NBO's efficacy for particular baby and parent groups and to pinpoint the elements of timing and duration that influence these interventions' efficacy. However, Barlow et al. [13] conducted a systematic analysis using the Cochrane technique to evaluate the impact of the NBAS and NBO systems on enhancing caregiver-infant attachment and related outcomes in both caregivers and babies.

Pediatric evaluation of disability inventory

In 1992, the pediatric evaluation of disability inventory (PEDI) was first made available to the public [14]. The PEDI was developed as a comprehensive assessment tool for inpatient pediatric rehabilitation programs. It was also designed to be used as an evaluation tool for outpatient therapy services, school programs, and community agencies that work with pediatric clients. Furthermore, a uniform protocol for disclosing functional disability in health policy data banks and data registries was developed. Finding out how much physical support the child usually receives from the caregiver, whether the child uses any modifications or adaptive equipment (such as braces or motorized wheelchairs), and what level of functional skills the child typically possesses are the three main goals of the assessment [15].

Over the past 10 years, the PEDI has played a crucial role in changing the way people think about child development, shifting the focus from a developmental perspective to a functional one. The PEDI has been utilized by academics and clinicians globally to emphasize discrepancies in the development of functional skills, underscore the significance of acknowledging cultural disparities, and emphasize the need to document functional progress in connection to therapies. PEDI has a long-standing history of contributing to the documentation of functional development. Proposed are novel approaches for the upcoming iteration of the PEDI, which involve the utilization of item banks and computer adaptive assessment. The new PEDI, with its computer adaptive testing feature and rewritten and enlarged material, is currently approved for clinical practice. This updated version allows therapists to assess children's functioning more quickly across a wider age range [14].

Child neuropsychological battery second edition

The child neuropsychological battery second edition (NEPSY II) is the updated version of the NEPSY, a developmental neuropsychological assessment that was initially published in 1998. The NEPSY assessment evaluated children between the ages of 3.0 and 12.11 in five areas of functioning: attention/executive function, language, sensorimotor, visuospatial, and learning/memory. The domains were derived conceptually rather than statistically. The NEPSY offered the benefit of co-normed subtests, enabling the comparison of scores within a test profile [16]. Recently, Zilli et al. [17] examined the neuropsychological characteristics and susceptibilities of children with epilepsy who do not have an intellectual disability or borderline intellectual functioning. Children diagnosed with epilepsy exhibited notable deficiencies in attention, executive functions, and sensorimotor skills as compared to the control group. Significant challenges were identified in social perception activities that include the identification of emotions, a skill that has been largely overlooked in children with epilepsy. The findings emphasize the significance of conducting a thorough assessment of cognitive abilities, specifically social cognition processes, in children with epilepsy who have average intellectual capacity. This is crucial for developing suitable interventions that aim to mitigate the long-term impact on educational and behavioral outcomes [17].

Vineland adaptive behavior assessment scale second edition

The Vineland adaptive behavior scales (VABS) were created in 1984 by Sparrow et al. and are extensively utilized in clinical and research contexts to assess the adaptive behavior of individuals with autism spectrum disorder [18]. Previous studies have shown that when children with autism spectrum disorder are compared to their non-autistic counterparts, who are the same age and mental age, they typically display deficiencies in their adaptive behaviors [19,20]. Then came the introduction of the VABS second edition of 2005 (Vineland II). This was useful in assessing the abilities of people with autism spectrum disorder because the specific social and communication difficulties that these children present with, along with the co-occurring behavioral issues associated with the disorder, made it difficult to accurately assess intelligence using standardized tools [21].

To evaluate concurrent validity, Scattone et al. [22] compared the Vineland II and BSID III norms. In support of the Vineland research, Yang et al. [23] found a distinct autism profile in scores comparable to Vineland II but not in standard scores. The cognitive scores on the two tests were the same, according to the authors, but the mobility and communication composite scores on the Vineland II were much higher.

Leiter international manipulative scale revised

Without requiring verbal communication, the Leiter international performance scale revised (Leiter-R) is a standardized assessment tool that assesses cognitive ability in people ranging in age from two years and 0 months to 20 years and 11 months. The Leiter-R's activities, which usually need a matching or pointing response, are made to be simple to understand and require minimal pantomimed instructions. The Leiter-R line of products includes foam shapes, cards, and stimulus easels. The test takes about 90 minutes to administer on average [24].

There are two batteries in the Leiter-R assessment: the attention and memory (AM) battery and the vision and reasoning (VR) battery. There are ten subtests in every battery. Not every age group is given access to every subtest, though. Depending on the particular therapeutic need, the VR and AM batteries can be used separately or in combination [24].

Basic cognitive functions like language, attention, memory, motor skills, and adaptive-social behaviors are evaluated on these exams. They are separated into subdomains related to development. The main difference between the Vineland II and PEDI scales is that they evaluate adaptive behavior [25].

Giofrè et al. [26] recently used a multigroup confirmatory factor analysis methodology to investigate the features of sex and gender differences using the Leiter-3, an entirely nonverbal cognitive assessment. The Leiter-3 was adapted for use in Italy. Studies show that although men and women may exhibit different traits, overall intelligence levels are similar for both sexes. Men do better than women on some tasks that require manipulating stimuli spatially, but not all activities. On the other hand, women consistently score better than men on tasks that call for more inhibition and attention control, such as the nonverbal stroop. The implications of our findings are of great clinical and practical significance. Men and women are known to have different cognitive capacities and limits, which emphasizes the need for tailored approaches in clinical assessments and interventions.

Selecting the best tool for children in India

A quick, low-cost, psychometrically sound instrument in Indian languages with items that are only developmentally or culturally appropriate is the perfect screening tool for Indian children. It has to be validated on a sample of healthy Indian children and needs little training. Since there is no designer tool like that, each physician has to decide what's best for their practice. Only two international development tools, namely, the parents' evaluation of developmental status (PEDS) ages and stages questionnaire and the assessment of developmental status, have been validated in Indian children [27]. Lately, few screening tools have been generated, taking international tools as standards.

India's usage of developmental screening tools

India uses a variety of screening methods, including those that are generated domestically in India, those that are translated into Indian languages, and those that have been developed and validated in high-income countries. Each category has a unique set of problems. Furthermore, techniques that have become well-known worldwide might not be suitable for our communities if they incorporate aspects that are new from a cultural perspective or that become meaningless when translated. Furthermore, to corroborate the results, a sizable number of healthy youngsters from the target group who are free of conditions like iron deficiency anemia, malnutrition, poverty, and lack of stimulation must be included [28].

Translations can be comprehensible, but they still face the aforementioned restrictions until they are confirmed. Indian instruments are linguistically and culturally suitable and have been validated, but they may not have ideal psychometric properties due to their initial development largely for community surveys conducted by health workers. A compendium of screening instruments for developmental delay that are regularly used or validated in Indian contexts was constructed, taking into account these considerations [27].

The most common instrument in India for validating and supporting new screening methods for assessing infants' and toddlers' development is the developmental assessment scale for Indian infants (DASII). This is an Indian version of the widely accepted international standard, the BSID. Nonetheless, there is disagreement amongst the various research studies carried out in India throughout the past 20 years on the precise DASII cutoff points that are employed to classify growth and differentiate between normal and unhealthy development [29].

Baroda developmental screening test

The Baroda developmental screening test (BDST) is a tool used to evaluate the motor and mental development of newborns. It was created by choosing certain questions from the BSID. The Baroda norms are a straightforward and efficient test that may be used by health workers during door-to-door surveys. The technique has a claimed sensitivity of 65% and a specificity of 95% [30].

Kishore et al. [31] conducted a comparison between the BDST, DST, and DASII full scales (motor and mental). A total of 30 infants, ranging in age from one to six months, who were receiving inpatient and outpatient perinatal mental therapy, were included in the study. Infants underwent screening using the DST and BDST tests, and their scores were evaluated using the DASII full-scale assessment. There is a strong correlation between DST, BDST, and DASII; however, only BDST can accurately predict scores. To establish the ability of BDST to identify developmental delays in infants born to women with prenatal psychiatric diseases, it is necessary to conduct studies with larger sample sizes and in various settings.

Trivandrum developmental screening chart

Nair et al. [32] developed and validated a simple evaluation tool to identify developmental delays in children aged 0-6 years in the community. The Trivandrum development screening chart (TDSC) is a tool used to assess the development of children between the ages of 0 and 6. It has a total of 51 items. These items were obtained from the standards identified in several developmental charts and scales. Professionals thoroughly assessed facial credibility and content credibility during the development of the TDSC. The criterion validity was assessed in a community sample of 1,183 children between the ages of 0 and 6 years, with an average age of 35.38 months (standard deviation of 19.25). The sample comprised 597 males, accounting for 50.46% of the total, and 586 females, representing 49.54%. The TDSC (0-6 years) was verified by comparing it to the Denver developmental screening test, which served as the standard for evaluation. When a delay in one item within the TDSC (0-6 years) was classified as "TDSC delay" (indicating a positive test result), the sensitivity and specificity of the TDSC (0-6 years) were determined to be 84.62% and 90.8%, respectively. The test-retest and inter-rater reliability, as assessed by the interclass correlation (ICC), showed a strong level of agreement, with a test-retest ICC of 0.77 and an inter-rater ICC of 0.97. The TDSC (0-6 years) is a reliable and precise assessment tool that can be utilized in the community to identify children between the ages of 0 and 6 who are experiencing developmental delays. This allows for the introduction of early intervention measures [32].

Indian Council of Medical Research psychosocial developmental screening test

The Indian Council of Medical Research (ICMR) developmental screening is utilized for evaluating psychosocial development, while the modified WHO parental interview schedule is employed for assessing family and micro-environmental factors. The assessment evaluates aptitudes in five domains of growth, including gross motor capabilities, visual and fine motor skills, auditory language and conceptual development, and social and personal skills [33].

In 1994, Vazir et al. [34] conducted a multi-centric cross-sectional collaborative study in three Indian centers to create the ICMR developmental screening. The goal was to establish straightforward and dependable indicators for identifying developmental disabilities in children under six, as well as to compare the timing of developmental milestones across the three regions. A psychosocial developmental screening test battery was created in the study, which was both affordable and culturally suitable. This instrument was then standardized on a sample of more than 13,000 children from rural, tribal, and urban communities in three regions of India. Inter-rater and test-retest reliability were employed to ensure the quality of the data. A total of 66 culturally suitable milestones were selected from a larger set of items during the pre-testing phase. The 50th percentile age reference values of the children in the Hyderabad study and the other two centers were comparable. In a later study conducted in 1998, Vazir et al. [35] found that children who were malnourished achieved developmental milestones at a delayed stage. Malnourished individuals had significant developmental delays, particularly in areas such as visual and fine motor skills, language and cognition, and personal social abilities. The delay ranged from seven to 11 months across different age groups in these places. Active paternal engagement in childcare, particularly by spending quality time with the kid, sharing tales, and bringing them on outings, has been identified as crucial for fostering optimal psychosocial development. Additional notable characteristics encompassed parental instruction, limited family size, and paternal career. The criteria that showed a significant correlation with improved nutritional quality in children were the child's hunger, absence of health issues, parental age, and the family's ownership of a house and access to electricity. Several studies have shown the efficacy of the ICMR psychosocial developmental screening test [36,37].

## Conclusions

With time, scales have undergone updates, as documented in the literature. Efforts have been undertaken to establish developmental standards for children in India. The presence of cultural differences in gender roles and self-care obligations is a challenge when it comes to administering international scales to Indian children. To promote psychosocial development in infants in developing nations, it is crucial to raise parental awareness and create demand for postnatal growth interventions. This requires appropriate identification of opportunities, selection of tools, acquisition and administration of these interventions, scoring and interpretation of data, and training in counseling to establish routine screening.
